# A Clinical Study of Precision Chemoablation for Hypertrophic Obstructive Cardiomyopathy Without Large Interventricular Septal Branches

**DOI:** 10.1002/clc.70095

**Published:** 2025-02-24

**Authors:** Xinnan Cao, Rong Huang, Haihua Geng, Hu Liu, Yi Gu, Peishu Xu, Yufeng Chen, Jian Yao, Hongzhuan Sheng

**Affiliations:** ^1^ Department of Cardiology, Affiliated Hospital of Nantong University Medical School of Nantong University Nantong China; ^2^ Department of Histology and Embryology Medical School of Nantong University Nantong China

**Keywords:** cardiomyopathy, gelatin sponge, hypertrophic, interventricular septal branches, percutaneous transluminal septal myocardial ablation, ultrasonic cardiogram

## Abstract

**Objective:**

This study aims to evaluate the clinical efficacy and safety of ultrasound‐guided percutaneous septal precision chemical ablation in the treatment of hypertrophic obstructive cardiomyopathy (HOCM).

**Methods:**

From December 2020 to July 2024, 27 patients with HOCM without large target septal branches (diffuse multiple branches, all less than 1 mm in diameter) were enrolled and underwent ultrasound‐guided percutaneous septal chemical ablation. Intraoperative left ventricular outflow tract gradient (LVOTG), postoperative cardiac troponin I (cTnI), complications, and changes in the 36‐Item Short Form Survey (SF‐36) score, New York Heart Association (NYHA) functional classification and echocardiography parameters in 1 year post‐PTSMA were monitored and analyzed.

**Results:**

Immediate postoperative LVOTG values monitored by catheter and echocardiography were both significantly decreased (both *p* < 0.05) in the 27 patients, whereas the cTnI level was increased after PTSMA treatment (*p* < 0.05). One patient developed transient complete right bundle branch block during the procedure. At the 1‐year follow‐up, these patients showed significantly increased scores in all the eight domains of the SF‐36 scale, and markedly improvement in echocardiography‐based LVOTG value (*p* < 0.05) and NYHA functional classification (*p* < 0.05). However, no significant change were observed in the mean interventricular septal thickness (IVSTh) and left ventricular ejection fraction (LVEF) before and after operation (*p* > 0.05).

**Conclusion:**

Ultrasound‐guided precision PTSMA with gelatin sponge is a safe and effective treatment approach for HOCM patients, which can reduce the left ventricular outflow tract obstruction and greatly improve the life quality of the patients.

## Graphical abstract

For HOCM patients without large target septal coronary arteries, we identify target septal coronary artery supplying the most hypertrophied septum, and make sure the guide‐wire can access this target vessel. We perform precise ablation of the most hypertrophied septum using gelatin sponge as an embolic medium.

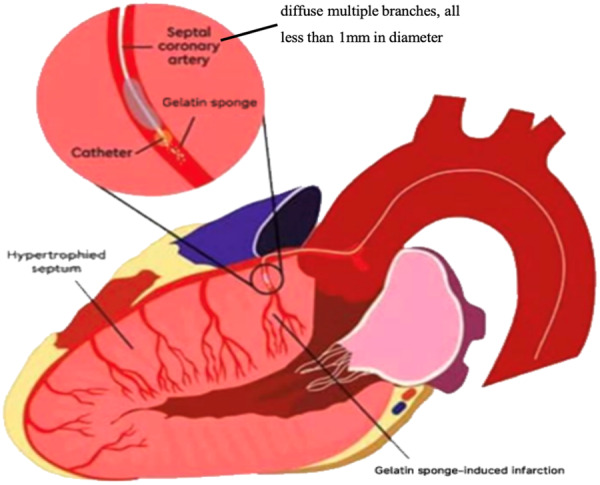

## 1 Introduction

Hypertrophic cardiomyopathy (HCM) is a common inherited cardiomyopathy characterized by asymmetrical hypertrophy of the interventricular septum. HCM is classified as obstructive or nonobstructive according to whether the resting left ventricular outflow tract gradient (LVOTG) is greater than 30 mmHg. For patients with hypertrophic obstructive cardiomyopathy (HOCM), the treatment options include medications, hypertrophic myocardial resection, and medical intervention [1, 2].

Percutaneous transluminal septal myocardial ablation (PTSMA) is the most common medical interventional approach [3] that requires relatively large target septal artery branches (>1 mm in diameter) [3, 4] and is usually performed with anhydrous alcohol as the medium [5]. At our hospital, we found that a large percent of HOCM patients had multiple diffuse septal branches with diameters less than 1 mm. However, the feasibility and efficacy of PTSMA in this group of patients have not been reported. In this study, we compared the intraoperative, postoperative, and 1‐year follow‐up data of 27 HOCM patients without large interventricular septal branches who received precision ablation of septal branch under echocardiography guidance at our hospital between December 2020 and July 2024 to assess the clinical efficacy and safety of PTSMA.

## 2 Data and Methods

### 2.1 Research Objectives

Twenty‐seven HOCM patients who received PTSMA the Affiliated Hospital of Nantong University between December 2020 and July 2024 were recruited in this study based on the clinical inclusion and exclusion criteria in the 2020 AHA/ACC guidelines [1]. Of the 34 patients, seven had large target septal branches and were excluded. A final total of 27 HOCM patients with multiple diffuse septal branches, without large target septal branches (<1 mm in diameter), and who had completed 1 year of follow‐up were included in this study.

This study was approved by the Ethics Committee of the Affiliated Hospital of Nantong University (2019‐K021‐01). All enrolled patients have signed the informed consent form.

### 2.2 Operation Method

Contrast agent was injected unilaterally into the radial artery for angiographic visualization of the coronary artery and left ventricle, and LVOTG was continuously monitored using a pigtail catheter. Left ventriculography was performed to confirm HOCM and to identify septal branches that supply the hypertrophic myocardium (target septal branches supplying the hypertrophic myocardium display myocardial bridge‐like changes in the angiogram). The guide wire was threaded through the guide catheter into the identified target septal branches. Once the OTW balloon was in place, the myocardial acoustic contrast agent hexavalentine was injected through the central cavity of the balloon, and sulfur hexafluoride was accumulated in the most hypertrophic part of the myocardium under ultrasound. If it was, the OTW balloon was used to block the septal branch, and LVOTG was observed under ultrasound. If the pressure decreased, gelatin sponge was injected into the central cavity of the OTW balloon for ablation. If the LVOTG decreased by less than 50% after ablation, the guidewire was further sent to the other septal branches in the proximal or distal segment according to the relationship between the hypertrophic myocardium and the ventricular septal branch, and the above steps were repeated until the LVOTG decreased by more than 50% compared with preoperative LVOTG or LVOTG was less than 30 mmHg.

### 2.3 Data Collection

Baseline clinical data were collected from the enrolled patients. The intraoperative target septal branches were determined, and the volume of gelatin sponge used was recorded. The preoperative and immediate postoperative LVOTG were compared and analyzed, and peak value of cardiac troponin I (cTnI) was recorded. The occurrence of complications was also observed. During the follow‐up period, SF‐36 score and changes in New York Heart Association (NYHA) functional classification were recorded. Changes in LVOTG, interventricular septal thickness (IVSTh), and left ventricular ejection fraction (LVEF) were also measured by echocardiography.

### 2.4 Statistical Analysis

Data were analyzed using SPSS26.0. Measured data are expressed as mean ± SD, median, and range. Count data are expressed as percentage (rate). Date are assessed using paired *t*‐tests of the preablation and postablation values. A *p* < 0.05 was considered statistically significant.

## 3 Results

### 3.1 Preoperative Clinical Features

The 27 patients had a mean age of 61.65 ± 23.62 years, and there were 11 males and 16 females. Comorbidities included hypertension in (*n* = 17, 62.96%), diabetes mellitus (*n* = 4, 14.81%), coronary artery disease (*n* = 9, 33.33%), and cardiac arrhythmia (*n* = 9, 33.33%). All 27 patients were adequately treated with medication that was either ineffective or intolerable, and were exhibiting preoperative clinical symptoms, predominantly chest tightness, chest pain, and shortness of breath. The clinical baseline data of the patients are shown in Table 1.

**TABLE 1:** Baseline clinical characteristics of 27 patients undergoing therapy for hypertrophic obstructive cardiomyopathy.

Age (year)	61.65 ± 23.62
Range/Median	53.00–70.00/60.00
Gender	
Males	11.00 (40.74%)
Females	16.00 (59.26%)
BMI	25.57 ± 2.60
Range/Median	23.39–28.09/25.09
Comorbidities	
Hypertension	17.00 (62.96%)
Diabetes mellitus	4.00 (14.81%)
Coronary artery disease	9.00 (33.33%)
Cardiac arrhythmia	9.00 (33.33%)
Medication	
Beta‐blockers	21.00 (77.78%)
Calcium antagonists	6.00 (22.22%)
NYHA classification	2.59 ± 0.56
Range/Median	2.00–3.00/3.00
Echocardiographic parameters	
IVSTh (mm)	18.37 ± 3.79
Range/Median	16.00–21.00/17.00
LVOTG (mmHg)	101.59 ± 17.04
Range/Median	89.00–111.00/100.00
LVEF (%)	0.60 ± 0.05
Range/Median	0.57–0.63/0.59
LA (mm)	43.81 ± 5.97
Range/Median	40.00–49.00/44.00
LVPW (mm)	12.30 ± 2.02
Range/Median	10.00–13.00/12.00

Abbreviations: BMI, body mass index; IVSTh, interventricular septal thickness; LA, left atrium; LVEF, left ventricular ejection fraction; LVOTG, left ventricular outflow tract pressure gradient; LVPW, left ventricular posterior wall; NYHA, New York Heart Association.

### 3.2 Operation

The mean number of septal branches in the 27 patients was 8.41 ± 1.42. Ablation was performed on the first septal branch in nine cases, on the second septal branch in five cases, on the third septal branch in eight cases, on the fourth septal branch in four cases, and on the fifth septal branch in one case. The mean volume of absorbable gelatin sponge injected was 0.64 ± 0.21 mL. The immediate postoperative LVOTG monitored by echocardiography (40.22 ± 11.40 mmHg vs. 101.59 ± 17.04 mmHg; *p* < 0.05) and by catheter (41.58 ± 26.30 mmHg vs. 103.04 ± 34.71 mmHg; *p* < 0.05) were significantly lower than the preoperative values. Postoperative cTnI level was significantly higher than the preoperative level (3.88 ± 1.23 ng/mL vs. 0.03 ± 0.01 ng/mL; *p* < 0.05). (Table 2) The changes in parameters for specific intraoperative conditions of each patient in Table 3.

**TABLE 2:** Comparison of preoperative and postoperative conditions.

Indicator (*n* = 27)	Mean ± SD	Median	Range
Septal branch	8.41 ± 1.42	8.00	8.00–9.00
Gelatin sponge (mL)	0.64 ± 0.21	0.60	0.50–0.70
LVOTG monitored by echocardiography (mmHg)			
Preoperative	101.59 ± 17.04	100.00	89.00–111.00
Immediate postoperative	40.22 ± 11.40^a^	40.00	30.00–50.00
LVOTG monitored by catheter (mmHg)			
Preoperative	103.04 ± 34.71	100.00	78.00–130.00
Immediate postoperative	41.58 ± 26.30^a^	32.50	18.75–63.50
CTnI (ng/mL)			
Preoperative	0.03 ± 0.01^a^	0.03	0.02–0.03
Postoperative	3.88 ± 1.23	3.40	3.00–4.98

Abbreviations: 1 mmHg = 0.133 kPa; CTn I, cardiac troponin I; LVOTG, left ventricular outflow tract pressure gradient.

^a^ *p* < 0.05, compared with preoperative values within the same group.

**TABLE 3:** Changes in parameters for specific intraoperative conditions for each patient.

Patient (*n* = 27)	Septal branch			Gelatin sponge (mL)	LVOTG monitored by echocardiography (mmHg)		
	Number	Diameter	Target vessel		Preoperative	Immediate postoperative	1‐year postoperative follow‐up
1	9	Small	First	1.00	101.00	35.00	24.00
2	11	Small	Second	0.50	111.00	51.00	60.00
3	10	Small	Second	0.60	130.00	59.00	65.00
4	9	Small	Fifth	0.50	82.00	22.00	30.00
5	8	Small	Fourth	0.60	93.00	40.00	40.00
6	5	Small	Second	1.20	91.00	24.00	37.00
7	8	Small	First	0.50	107.00	51.00	54.00
8	7	Small	First	0.70	89.00	25.00	30.00
9	11	Small	Third	0.90	147.00	56.00	66.00
10	7	Small	Second	1.10	100.00	30.00	42.00
11	10	Small	Third	0.70	85.00	30.00	54.00
12	8	Small	Second	0.50	103.00	53.00	60.00
13	9	Small	Fourth	0.80	83.00	27.00	31.00
14	10	Small	Fourth	0.50	132.00	50.00	53.00
15	5	Small	First	0.50	81.00	31.00	28.00
16	8	Small	First	0.60	89.00	38.00	29.00
17	7	Small	Third	0.70	120.00	50.00	51.00
18	9	Small	Fourth	0.40	92.00	31.00	30.00
19	8	Small	First	0.50	102.00	47.00	45.00
20	8	Small	First	0.60	124.00	59.00	60.00
21	8	Small	Third	0.60	100.00	45.00	42.00
22	9	Small	First	0.40	86.00	31.00	35.00
23	9	Small	Third	0.50	116.00	48.00	46.00
24	9	Small	Third	0.60	89.00	32.00	37.00
25	8	Small	Third	0.40	82.00	27.00	40.00
26	9	Small	Third	0.80	99.00	45.00	51.00
27	8	Small	First	0.50	109.00	49.00	49.00

Abbreviations: 1 mmHg = 0.133 kPa; LVOTG, left ventricular outflow tract pressure gradient.

A complete right bundle branch block was observed in only one patient during the operation and was resolved after 4 h. Other types of arrhythmia or complications such as pericardial effusion, heart failure, or death were not observed.

### 3.3 Postoperative Follow‐Up

Clinical symptoms such as chest tightness, chest pain, and shortness of breath were significantly alleviated or disappeared after PTSMA. At 1 year after operation, the patients scored significantly higher in physiological functioning, physiological role limitation, physical pain, general health, vitality, social functioning, emotional role limitation, and psychological health in the SF‐36 than their preoperative scores (Table 4). In addition, NYHA classification was also significantly improved (Table 5; all *p* < 0.05).

**TABLE 4:** Comparison of SF‐36 scale scores for each dimension before and after operation.

Dimension (*n* = 27)	Preoperative			1‐year postoperative follow‐up			*p*
	Mean ± SD	Median	Range	Mean ± SD	Median	Range	
Physiological—function	48.70 ± 12.74	50.00	40.00–60.00	82.78 ± 5.33^a^	85.00	80.00–85.00	< 0.05
Physiological—role limitations	37.04 ± 18.47	25.00	25.00–50.00	70.37 ± 13.67^a^	75.00	50.00–75.00	< 0.05
Somatic pain	45.74 ± 15.09	41.00	41.00–62.00	76.81 ± 9.27^a^	74.00	74.00–84.00	< 0.05
Overall health	40.41 ± 7.75	40.00	35.00–47.00	69.48 ± 5.93^a^	72.00	67.00–72.00	< 0.05
Vigor	39.44 ± 13.49	45.00	25.00–50.00	75.19 ± 6.53^a^	75.00	70.00–85.00	< 0.05
Social—functioning	57.41 ± 14.13	62.50	50.00–62.50	84.72 ± 7.86^a^	87.50	75.00–87.50	< 0.05
Emotional role—limitations	40.74 ± 22.85	33.30	33.30–66.70	76.57 ± 15.21^a^	66.70	66.70–100.00	< 0.05
Mental health	55.23 ± 16.03	56.00	36.00–64.00	75.70 ± 10.83^a^	76.00	76.00–80.00	< 0.05

Abbreviation: SF‐36, 36‐entry health survey short form.

^a^ *p* < 0.05, comparison of preoperative and 1‐year postoperative follow‐up within the same group.

**TABLE 5:** Changes in NYHA classification and echocardiographic parameters preoperatively and at follow‐up.

Indicator (*n* = 27)	Preoperative			1‐year postoperative follow‐up			*p*
	Mean ± SD	Median	Range	Mean ± SD	Median	Range	
NYHA classification	2.59 ± 0.56	3.00	2.00–3.00	1.37 ± 0.48^a^	1.00	1.00–2.00	< 0.05
LVOTG (mmHg)	101.59 ± 17.04	100.00	89.00–111.00	44.04 ± 12.11^a^	42.00	31.00–54.00	< 0.05
IVSTh (mm)	18.37 ± 3.79	17.00	16.00–21.00	16.73 ± 3.07	15.00	15.00–18.00	> 0.05
LVEF (%)	0.60 ± 0.05	0.59	0.57–0.63	0.62 ± 0.04	0.62	0.60–0.64	> 0.05

Abbreviations: 1 mmHg = 0.133 kPa; IVSTh, interventricular septal thickness; LVEF, left ventricular ejection fraction; LVOTG, left ventricular outflow tract pressure gradient; NYHA, New York Heart Association.

^a^ *p* < 0.05, comparison of preoperative and 1‐year postoperative follow‐up within the same group.

Echocardiography at 1 year post‐PTSMA revealed markedly decreased LVOTG (*p* < 0.05), but no significant changes in IVSTh and LVEF (both *p *> 0.05) (Table 5).

## 4 Discussion

Hypertrophic obstructive cardiomyopathy often causes discomfort such as dyspnea, chest pain, fatigue, and dizziness [1, 3]. For patients with poor response to medication and cannot or unwilling to undergo surgery, medical intervention is recommended. The most common type of such intervention is PTSMA, and anhydrous alcohol is the most classic medium for chemical ablation [5]. Suitable septal branch arteries (>1 mm in diameter) need to be selected as the target vessels during the procedure [3, 4], and chemical ablation of large septal branches by anhydrous alcohol can effectively eliminate the obstruction. However, it has not been reported whether precise ablation of the septal branches supplying the hypertrophic ventricular septum can achieve ideal ablation effect when the septal branches are diffuse and multiple relatively small vessels. Thus, the aim of this study was to evaluate the clinical efficacy and safety of ultrasound‐guided percutaneous septal precision chemical ablation in the treatment of hypertrophic obstructive cardiomyopathy (HOCM).

Since gelatin sponge is highly viscose, it is safer than anhydrous alcohol and prevents myocardial infarction in nontarget regions [6, 7]. In this procedure, gelatin sponge was used as the ablation medium to precisely ablate the septal branches supplying the most hypertrophic myocardium.

Previous studies and guidelines have suggested that the immediate success of percutaneous transluminal septal chemical ablation for HOCM is defined as a ≥50% reduction in LVOTG [3, 5]. Catheter monitoring and echocardiography revealed that LVOTG was decreased by more than 50% immediately postablation, respectively, which suggests that gelatin sponge‐based precision ablation is efficacious for HOCM with tiny septal branches.

SF‐36 is a reasonable, relatively short and simple scale that not only measures the health status of people of all ages, but also assesses the quality of survival of patients with various diseases in the clinical setting [8, 9]. We used the SF‐36 to assess the quality of survival of patients with HOCM following operation and found that the patients scored higher in all eight domains of SF‐36, including physiologic function, somatic pain, general health, and so on in the postoperative period. Furthermore, NYHA classification was decreased and symptoms such as chest tightness and shortness of breath were significantly improved after operation.

Echocardiography revealed that LVOTG was significantly reduced at 1 year after PTSMA. Moreover, the improvements in clinical symptoms and decreased LVOTG in patients who were followed to 1 year after PTSMA suggested that precision ablation is effective for HOCM with tiny target septal branches. This may be attributed to the attenuation of myocardial movement by precision ablation at the site of most severe hypertrophy. However, the long‐term effect of this treatment warrants further investigation. Moreover, echocardiography at 1 year post‐PTSMA showed no significant change in septal thickness in HOCM patients, which may be related to the small extent of myocardial infarction caused by precision ablation of the target septal branches.

PTSMA may cause serious complications, such as ventricular tachycardia, ventricular fibrillation, and septal perforation, and 10%–15% of patients require implantation of a permanent pacemaker due to atrioventricular block [8, 10]. In addition, severe intraoperative drop in blood pressure or acute left heart failure resulting in death have also been reported [2]. In this study, only one patient developed a complete right bundle branch block, which was resolved at 4 h after the procedure. There were no other complications or deaths. These findings may be related to the high safety profile of the modified precision ablation procedure at our center, since precision ablation of the target septal branches and the use of gelatin sponge as medium prevented extensive myocardial infarction in nontarget regions. Given that precision ablation has a low incidence of complications, temporary pacemakers are no longer installed when performing precision ablation for HOCM, reducing patient's pain and medical costs.

At the 4‐month postoperative follow‐up, three patients showed significantly elevated LVOTG and subsequently underwent a second ablation of the target septal branches. The efficacy of the procedure was confirmed at the 1‐year follow‐up. Since PTSMA is less invasive and relatively inexpensive, multiple ablations can be performed according to LVOTG changes to achieve optimal outcome in HOCM patients with multiple diffuse septal branches.

Our study demonstrated that gelatin sponge‐based precision ablation is an effective treatment with minimal complications for HOCM patients without large target septal branches. Thus, this study expands the population of HOCM suitable for chemical ablation. However, since this was a single‐center study with a small sample size and a follow‐up period of only 1 year, the long‐term effects and safety of this treatment will warrant further investigation.

## Author Contributions

Xinnan Cao and Rong Huang designed the study and drafted the manuscript, Xinnan Cao, Rong Huang and Haihua Geng performed literature search and statistical analysis of the data, Hu Liu, Yi Gu, Peishu Xu, Yufeng Chen and Jian Yao provided subject matter expertize in hypertrophic cardiomyopathy to identify strengths and weaknesses of the methods of this study, Hongzhuan Sheng was responsible for the integrity of the work from inception to publication. All authors read and met the criteria for authorship and agreed with the results and conclusions of the report.

## Ethics Statement

This study was approved by the Ethics Committee of Nantong University Hospital, Jiangsu Province, China. All enrolled patients signed an informed consent form.

## Conflicts of Interest

The authors declare no conflicts of interest.

## Data Availability

All datasets generated for this study are included in the article. The data that support the findings of this study are available from the corresponding author upon reasonable request.
